# Biogeography of Argentine Stem Weevil Invasion of Southern New Zealand and Two Offshore Islands

**DOI:** 10.1002/ece3.74483

**Published:** 2026-11-03

**Authors:** S. L. Goldson, P. K. Dearden, K. R. Dainty, S. N. Inwood, M. R. McNeill, J. W. M. Marris, F. Tomasetto, B. I. P. Barratt

**Affiliations:** ^1^ Bioeconomy Science Institute Tuhiraki Lincoln, Christchurch New Zealand; ^2^ Bioprotection Aotearoa, Genomics Aotearoa, and Biochemistry Department University of Otago Dunedin New Zealand; ^3^ Department of Pest‐Management and Conservation Lincoln University Lincoln New Zealand; ^4^ Bioeconomy Science Institute, Invermay Research Centre Mosgiel New Zealand

## Abstract

Understanding the biogeography and genetics of the invasion of oceanic islands by pest species can provide insights into how to limit such incursions. The invasive pasture pest weevil, *Listronotus bonariensis* (Kuschel), has established in both New Zealand and some of its offshore islands, causing significant economic damage. The opportunity was therefore taken to compare both the species' morphology and genetics of populations collected from the New Zealand mainland, Stewart Island (30 km offshore) and Chatham Island (860 km offshore). The *L. bonariensis* individuals from both of the offshore populations were found to be significantly smaller than those of the mainland populations. However, based on classical and molecular taxonomy, no associated species‐level differences were found and it was concluded that the island's smaller populations were the result of relatively poor habitat quality. Further, no significant genetic differences were detected between Stewart Island and mainland populations, indicating a close connection. Conversely, at the genomic level, the Chatham Island population was significantly different from the two other populations and this was attributed to probable founder effects. The evidence points to a small subset of what was still a recently‐established New Zealand *L. bonariensis* population early in the 20th century being inadvertently transported to the Chatham Islands. Thereafter, this founding subset expanded such that more recent introductions have had little effect on the founding genetics of weevils on the island. This study investigates whether irregular, unintended human transportation of species to isolated islands can lead to the occupation of previously uninhabited niches, with founder effects leading to genetic divergence from conspecific source populations elsewhere.

## Graphical abstract

This is a study that covers the biogeography and genetics of the invasive pasture stem weevil *Listronotus bonariensis* on the New Zealand mainland and two off‐shore islands. This considers the insect's morphology and genome in all locations and shows a founder effect.

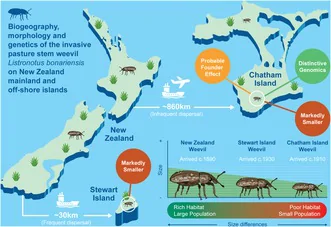

## 1 Introduction

Studies of island biogeography provide valuable insights into the understanding of evolution (MacArthur and Wilson 2001; Whittaker et al. 2017). Such work shows how evolution shapes invasive island populations and how the selective pressure of new environments affects form and function (Brener and Cockell 2025). Therefore, speciation on ‘oceanic island archipelagos’ has been well studied. Examples of such work have been conducted in the Galapagos Islands (Andrus et al. 2009; Benavides et al. 2009; Peck 2006), Hawaii (Gruner et al. 2008; Price and Clague 2002) and Indonesia (Walton et al. 1997). The mechanisms driving such island speciation include the impacts of geographical isolation in Hawaii (e.g., Hembry et al. 2021) unique selection pressure (e.g., Matsubayashi et al. 2010), ecological release (e.g., Herrmann et al. 2021) and founder effects (e.g., Emerson et al. 2025).

Dispersal of pest species provides interesting perspectives on island biogeography (Gaston et al. 2003; Gillespie and Roderick 2002). Species, arriving both deliberately or accidentally, often coincide with changing environments resulting from land use changes (Meijer et al. 2011; Sánchez‐Ortiz et al. 2020; Zhao et al. 2024). Over short intervals, such incursions can provide key information as to how species respond adaptively to the novel invaded environment.

New Zealand, with its c. 600 widely dispersed and diverse isolated island ecosystems, provides an exceptional opportunity to study the genetics and ecology of invasions by exotic species and their introduced biological control agents. A recent New Zealand example of this is a study by Hussain et al. (2025), who, through whole‐genome sequencing of the Irish parthenogenetic strains of the parasitoid *Microctonus aethiopoides* Loan (Hymenoptera: Braconidae), were able to assess the species' spatial and temporal variation and the genetic basis for their reproduction. Based on five populations, this work revealed patterns of high heterozygosity and short homozygous segments, which in turn supported the possibility of automictic thelytoky, probably through central fusion.

This contribution similarly presents findings on the morphology and genetics of ‘mainland’ and island populations of the severe grassland pest species Argentine stem weevil *Listronotus bonariensis* (Kuschel) (Coleoptera: Curculionidae) (Figure 1). This weevil established in mainland New Zealand at the turn of the 20th century (e.g., Stewart et al. 2023). Williams et al. (1994) ascertained that the species probably originated in the Buenos Aires/Uruguay area in South America. In view of the weevil's pest impact, its biology and ecology have been extensively researched (e.g., Pottinger 1961; Barker 1989a, 1989b; Barker et al. 1989a, 1989b; Goldson 1979; Goldson 1981; Prestidge and van der Zijpp 1985; Goldson et al. 1998; McNeill et al. 1998; Goldson et al. 2011; Goldson et al. 2020; Goldson et al. 2023; Vereijssen et al. 2024). In 1990, a parthenogenetic endoparasitoid of *L. bonariensis* , *Microctonus hyperodae* Loan (Hymenoptera: Braconidae), was introduced into New Zealand as a biocontrol agent of the weevil (Goldson et al. 1990) and widely released (Goldson et al. 1993). This led to the suppression of the pest (e.g., Barker and Addison 2006; Goldson et al. 1998; Barker 2013). Both *L. bonariensis* and *M*. *hyperodae* are now distributed throughout mainland New Zealand and on Chatham Island, 860 km east of the New Zealand mainland and Stewart Island, 30 km south of the New Zealand mainland (Figure 2).

**FIGURE 1:**
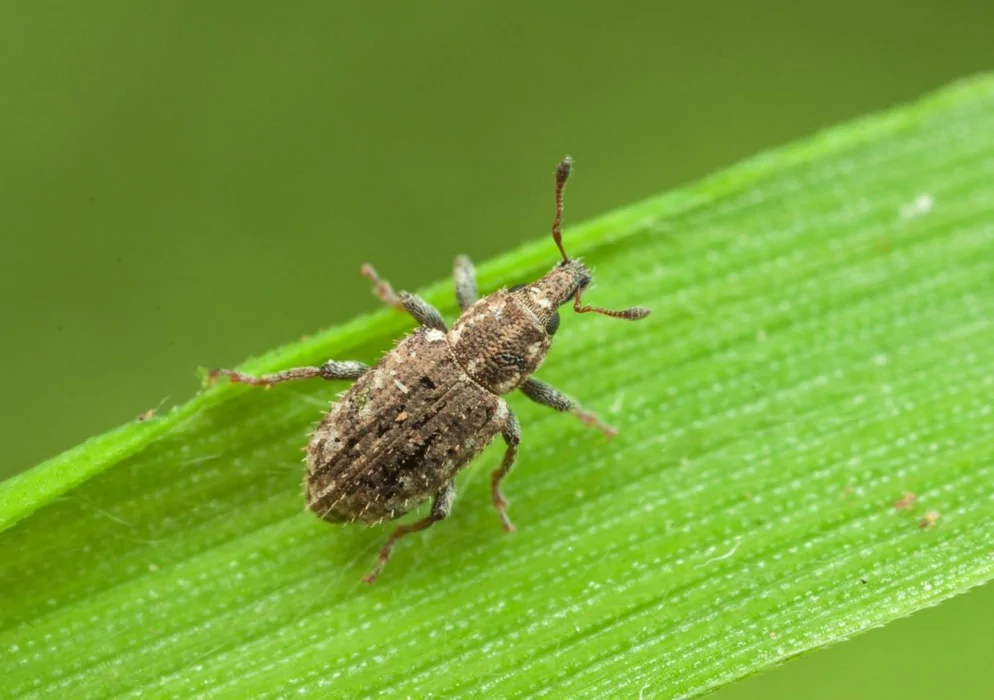
*Listronotus bonariensis* (Kuschel). Photo credit by Bryce McQuillan.

**FIGURE 2:**
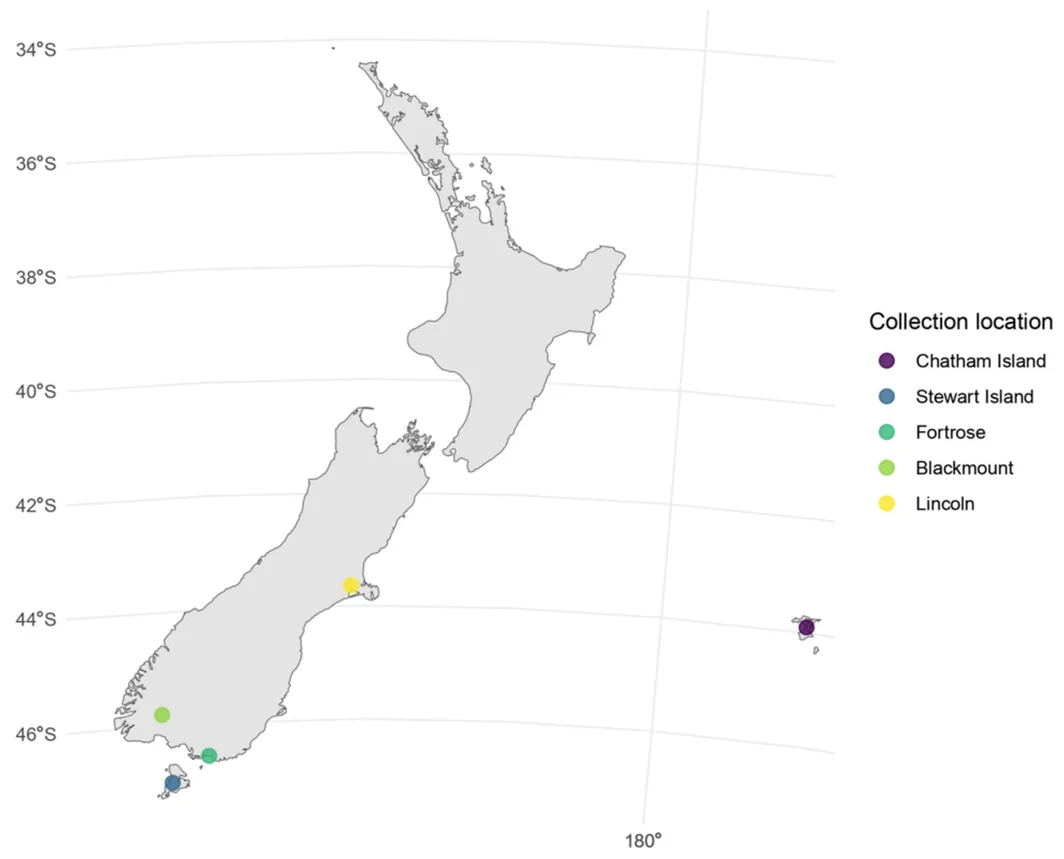
New Zealand sampling locations of *Listronotus bonariensis* and names as referred to in the text.

As part of the ongoing research into *L. bonariensis* , there has been relatively little investigation into the genomics of the species (Williams et al. 1994; Harrop et al. 2020; Inwood et al. 2024). Following assembly of an incomplete and fragmented draft reference genome for *L. bonariensis* , with a single‐copy BUSCO completeness of 78.8% and a minimum repeat content of 71.3%, Harrop et al. (2020) genotyped weevil populations using a reduced‐representation approach with genotyping‐by‐sequencing (GBS). Despite the poor genome assembly quality, this showed that the weevil has high levels of heterozygosity and low population structure consistent with a large effective population with frequent gene flow. Through site frequency spectra analysis of the GBS data, using different models of demographic history, Harrop et al. (2020) found the model with the best fit supported separate weevil population introductions from their South American origin into areas north and south of New Zealand's Main Divide, with subsequent admixing of the two. However, numerous questions remain relating to the distribution and genetics of the *L. bonariensis* populations across mainland New Zealand and its offshore island populations. More specifically, this study aimed to improve the understanding of the impacts of island invasion on the weevil's genomic diversity, body morphology and size.

## 2 Materials and Methods

### 2.1 Collection and Measurements of *Listronotus bonariensis* Samples for Phenotypic Measurements and Genotypic Analysis

To compare the overall size of adult weevils, 16 males and 16 females were examined from specimens collected from each of the Chatham Island in 2014, Stewart Island in 2016 and mainland New Zealand (Lincoln) in 2016, amounting to a total of 128 individuals (Figure 2). These were stored in 95% ethyl alcohol. In addition, elytral measurements were made from 30 pinned specimens obtained from the Lincoln University Entomology Research Collection, which were collected from low‐fertility marginal areas such as non‐irrigated, uncultivated roadside areas comprising mixes of low‐productivity grasses and weed species and often in native tussock areas. Such areas are more similar to the environments found on Chatham and Stewart Islands. For this paper, these areas are referred to as the ‘mainland marginal ecosystems’. Weevil specimens were necessarily collected opportunistically given the logistics of access to Chatham and Stewart Islands. The implications of such non‐contemporaneous sampling are considered in the Discussion.

The elytral lengths of the weevil populations were measured from the anterior margin of the scutellum to the apex of the elytra along the suture. Measurements were made using a binocular microscope fitted with an eyepiece graticule or electronically using the measurement function of the Nikon NIS‐Elements software.

For comparison of *L. bonariensis* reproductive structures, four male and four female weevils, respectively, were collected from the Chatham Islands in 2022 and a mainland southern area known as Blackmount (Figure 2) in 2023 and stored after collection in 95% ethyl alcohol. These weevils, representing contrasting regional populations, were dissected under a binocular microscope to remove the aedeagus (males) and the female sternite 8 (females). The genitalia were then cleared by soaking them in a 10% solution of KOH for 24 h and were then temporarily mounted in glycerol for measurement. The specimens were photographed using a digital camera attached to a dissecting microscope, and composite images were generated using Nikon NIS‐Elements software using the image stacking function and edited using Adobe Photoshop Elements (Figures S1 and S2).

Genotypic analysis was based on > 30 male and female weevils collected from each of the following locations: Chatham Island in 2014, Stewart Island in 2016, mainland Lincoln in 2016 and mainland Fortrose (Figure 2) in 2020. These weevils were stored at −40°C. All field specimens were collected from pasture foliage using a modified blower vac set on suction mode (Echo ES‐2400, 24 cc, Kioritz Corporation, Tokyo) (McNeill and van Koten 2020).

### 2.2 Statistical Analyses

#### 2.2.1 Elytral Lengths

The elytral lengths among weevil specimens collected from Chatham Island, Stewart Island, mainland Lincoln (Figure 3) and the mainland marginal ecosystems were analysed as follows. Each dataset was stratified by sex (two levels). To assess the effects of location on elytral length, a non‐parametric complete randomised permutation test was used (*n* = 10,000 cycles) to test for statistical interactions. The permutation probabilities were analysed using a factorial analysis of variance (ANOVA) implemented in the ‘lmPem’ R package. The ANOVA results, in conjunction with the permutation test, were used to derive *p*‐values. Post hoc statistical significance was determined using Tukey's HSD pairwise permutation tests, conducted within the ‘stats’ package in R 4.3.1 (R Development Core Team 2023).

**FIGURE 3:**
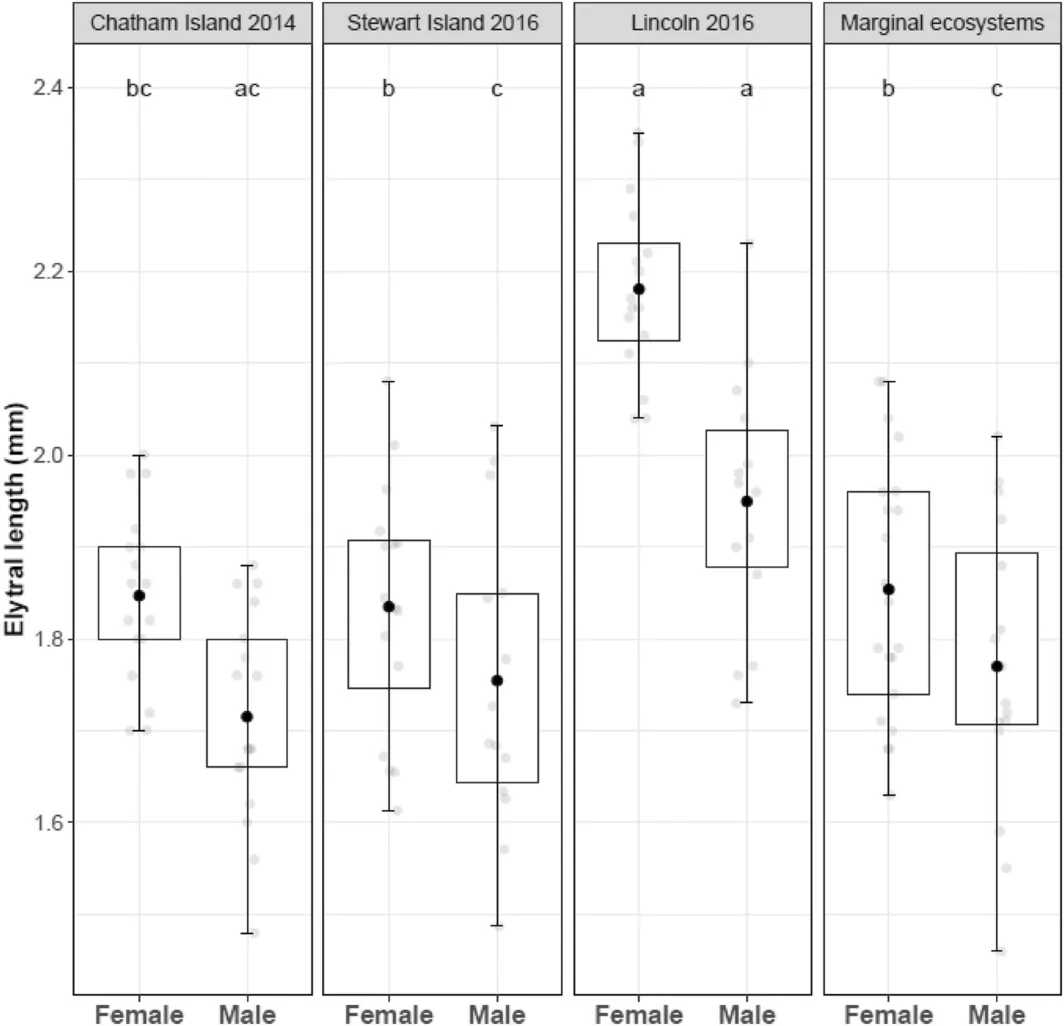
Boxplot for elytral lengths within male and female *Listronotus bonariensis* collected on the Chatham and Stewart Island and Lincoln (i.e., New Zealand mainland). The whiskers represent standard error of the mean (S.E.M). Means (black dots) with different letters were significantly different (*p* < 0.001) in pairwise comparisons.

#### 2.2.2 Reproductive Structures

Reproductive structures were closely examined for indications of phenotypic differences, and images were examined for further verification by Dr. Samuel D.J. Brown (Bioeconomy Science Institute, Auckland), a coleopterist with expertise in weevil morphology.

#### 2.2.3 Weevil Population Genotyping

##### 2.2.3.1 DNA Extraction

DNA was extracted from the heads of ten *L. bonariensis* samples collected from Stewart Island, Chatham Island, Fortrose and Lincoln (Figure 2) using a Quick‐DNA Tissue/Insect kit (Zymo Research Ltd) according to the manufacturer's protocol.

##### 2.2.3.2 DNA Analysis and Interpretation

Library preparation was performed with a DNA Prep Kit (Illumina) according to the manufacturer's protocol, using 100 ng of DNA input. The sequencing library was quality‐checked using a Qubit dsDNA Broad Range kit (Thermofisher) and run on a Tapestation with D5000 ScreenTape (Agilent). Samples were then whole‐genome sequenced on an Illumina NovaSeq 6000, generating 151 bp paired‐end reads. DNA extractions, library preparation and sequencing were performed by AgResearch, Invermay, New Zealand. This project used whole‐genome sequencing rather than the reduced representation GBS approach used by Harrop et al. (2020); however, resulting variants were also merged with those detected using GBS in a subsequent analysis.

Sequencing reads from *L. bonariensis* individuals were quality‐trimmed and had adapters removed with the BBTools v37.57 suite program BBDuk (https://archive.jgi.doe.gov/data‐and‐tools/software‐tools/bbtools/bb‐tools‐user‐guide). This was done using suggested parameters, including first trimming the raw reads to the nearest multiple of 5 bp to remove any extra bases beyond 150 bp with poorly‐calibrated quality scores, and with the suggested quality score filter increased to Q15. Reads were then indexed and mapped to the NCBI *L. bonariensis* reference genome GCA_014170235.1 assembled by Harrop et al. (2020) with the Burrows‐Wheeler Alignment tool (BWA) v0.7.17 (Li and Durbin 2009) using BWA‐MEM with default parameters. Files were then sorted and indexed with SAMtools v1.10‐98 (Danecek et al. 2021), with inspection revealing that mean coverage across all samples was 82.7% (Table S1). Bam files were converted to cram files using the NCBI *L. bonariensis* reference genome GCA_014170235.1 (Harrop et al. 2020). CRAM files were then sorted, and duplicate alignments were marked with SAMtools v1.10‐98. SNPs and indels were identified in samples of *L. bonariensis* against GCA_014170235.1 with GATK v4.3.0.0 HaplotypeCaller (McKenna et al. 2010) with ploidy set to two. Variants were then called for each sample by joint genotyping with GLnexus v1.4.1 (Lin et al. 2018) converted from gVCF files to VCF files and then indexed with BCFtools v1.16 (Danecek et al. 2021). Variants were filtered jointly across all samples, retaining biallelic sites with a minimum allele frequency of 0.05, an average GQ score above 20 and a QUAL score above 40, with sites excluded if the fraction of missing genotypes was more than 0.2, using BCFtools v1.16. As the reference assembly does not contain an identifiable mitochondrial genome, mitochondrial variation could not be assessed.

Tajima's *D* (Tajima 1989) was calculated using VCFtools v0.1.16 (Danecek et al. 2011) on quality‐filtered SNPs, using a non‐overlapping sliding window of 100 kb. PLINK2 v2.00a5.10 (Chang et al. 2015); this was used to calculate pairwise population differentiation (*F*_ST_) using the Weir & Cockerham method (Weir and Cockerham 1984).

PLINK2 v2.00a5.10 was then used to reduce linkage disequilibrium (LD) among quality‐filtered SNPs. This LD pruning used a sliding window approach with a 10 kb window size, an incremental step of 10 bp and an *r* ^2^ threshold of 0.1, using the flag –bad‐ld due to having fewer than 50 samples. Variants were retained if they showed low LD with other SNPs in the dataset. Plink v2.00a5.10 was then used to perform a principal components analysis (PCA) based on linkage‐pruned SNPs. Linkage‐pruned SNPs were also used to investigate admixture between populations using ADMIXTURE v1.3.0 (Alexander et al. 2009) with *K* values ranging from two to ten, performing cross‐validation testing and with a random seed set for reproducibility. The Clumpak web server (Kopelman et al. 2015) was then used to align cluster results across multiple *K* values to ensure consistent cluster identity in plots. For *K* values between two and four, each sample was assigned to the ADMIXTURE cluster for which it had the highest ancestry proportion. These ADMIXTURE‐inferred cluster assignments were then used in place of sample population to visualise PCA results.

Outputs from the above analyses were plotted in R v4.1.1 (R Development Core Team 2023) with pheatmap v1.0.12 (Kolde 2019) used to plot the FST results, and remaining plots generated using ggplot2 v3.4.0 (Wickham 2016) using the viridis v0.6.2 colour palettes (Garnier et al. 2021).

To combine these whole‐genome sequence data, the quality‐filtered SNP file was then intersected with the GBS SNPs to generate a VCF containing variants found in both datasets, using Tassel v5.2.96 (Bradbury et al. 2007; Glaubitz et al. 2014). This merged VCF was then filtered to remove sites where the fraction of missing genotypes was more than 0.2. PLINK2 v2.00a5.10 was again used to reduce linkage disequilibrium (LD) among quality‐filtered SNPs as above, and to then perform a PCA on the linkage‐pruned merged dataset.

Consensus FASTA files for each sample were generated from VCF files using BCFtools v.1.16 (Danecek et al. 2021) before Mashtree v1.2.0 (Katz et al. 2019) was used to calculate the mash distance of the sequenced individuals with a bootstrap of 100. The SplitsTree App 6.0.0 (Huson and Bryant 2024) was used to perform the Neighbour Net method (Bryant and Moulton 2004; Bryant and Huson 2023) with default options so as to obtain 48 splits, cyclic. The Show Splits method was then used to generate a Split Network visualisation with default options.

## 3 Results

### 3.1 Elytral Lengths

Significant phenotypic differences in elytral lengths were found between *L. bonariensis* specimens collected from mainland New Zealand and those from the Chatham and Stewart Islands. The female and male *L. bonariensis* elytral lengths in specimens from the 2016 mainland Lincoln sample were 1.95 ± 0.028 mm and 1.81 ± 0.025 mm, respectively. This result is consistent with Goldson (1979), who earlier reported the mean elytral length of female *L. bonariensis* from the Lincoln site collected in the 1977–78 season to be 2.053 ± 0.028 and males 1.872 ± 0.023. As with Goldson's (1979) Lincoln study, female elytral lengths in the 2016 population were significantly greater than those of the males (*p* < 0.001).

Figure 3 indicates that the elytral measurements of weevils from the Chatham Island, Stewart Island and the mainland marginal ecosystems were all significantly smaller than those collected at Lincoln, with no significant differences between the sexes. The female elytral lengths were 1.85 ± 0.02 mm, 1.83 ± 0.03 mm and 1.85 ± 0.03 mm, respectively, in weevils collected from Chatham Island, Stewart Island and the mainland marginal ecosystems; these therefore show close uniformity in size.

### 3.2 Reproductive Structures

Comparison of male and female genitalia from *L. bonariensis* specimens collected from mainland Blackmount and Chatham Island indicated no species‐level differences (Figures S1 and S2). Genitalia of both males and females of *Listronotus* spp. vary considerably between species and are reliable characters for species delimitation (O'Brien 1977). The aedeagus is typically used to distinguish between species within the same genus (Hönnicke et al. 2010). Published illustrations of North American *Listronotus* spp. (Henderson 1940; O'Brien 1977) demonstrate substantial species differences in the overall shape of the aedeagus, as well as the armature of the internal sac, both in the form of the ostial valves and the size and shape of the basal sclerites (using the terminology of Lindroth (1957)). There are also substantial differences in the sclerotisation and shape of the blade of female sternite 8 between species. The New Zealand populations of *L. bonariensis* from both Blackmount and Chatham Island (Figure S1) have a penis that is widest at the ostium, with a ventrally‐directed, strongly trapezoid‐shaped apex; large, crescent‐shaped ostial valves; and large, heavily sclerotised, U‐shaped basal sclerites (Goldson and Emberson 1981). Females from both populations have a similar shape to sternite 8, being straight‐sided but divergent towards the apex. The centre of the blade is thinly sclerotised and transparent, and the apex shows moderately sclerotised margins into which fine setae are inserted (S. Brown, pers. comm.). These features are diagnostic of *L. bonariensis* .

### 3.3 Genotypic Differences

Whole‐genome sequencing of 40 *L. bonariensis* individuals collected from Stewart Island, Chatham Island, Fortrose and Lincoln (Figure 2) generated an average of 94 million reads per sample, which resulted in satisfactory mapping rates, quality and genome coverage (Table S1). Following variant calling, filtration and linkage‐pruning, variation in the genomes was assessed using principal component (PC) analysis. Notably, on PC1 (representing 11.99%), individuals from Chatham Island clustered distinctly from those individuals collected from the two mainland sites and the Stewart Island population (Figure 4A). No other clear clustering or population stratification was identified across PC2–PC20 (Figure S3).

**FIGURE 4:**
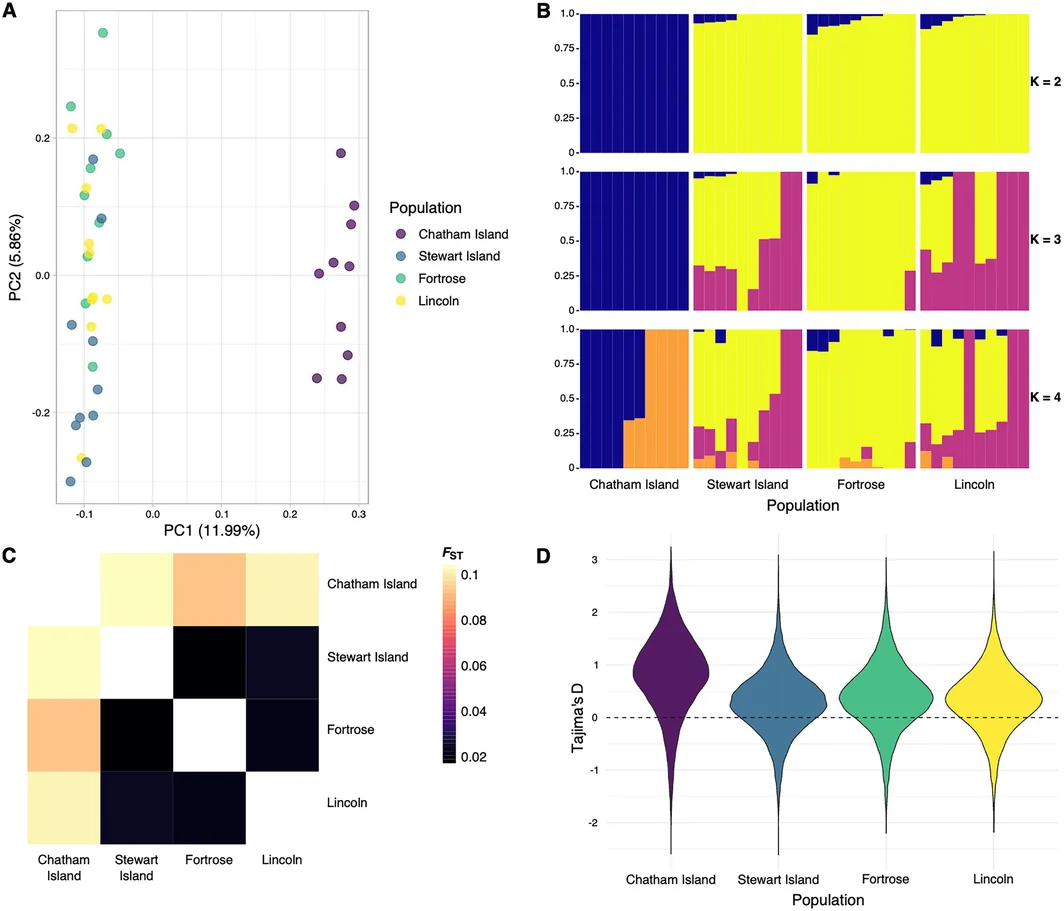
Genetic structure of *Listronotus bonariensis* populations. (A) Principal Component analysis (PCA) of 40 sequenced *L. bonariensis* genomes, based on 6,688,548 linkage‐pruned SNPs. Axes represent PC1 and PC2, colours represent collection location. (B) Population admixture analysis with *K* = 2 (top), *K* = 3 (middle) and *K* = 4 (bottom), where each colour represents a distinct genetic cluster and each vertical bar represents a different sample. The *K* value with the lowest cross‐validation error was 2. (C) A heatmap displaying pairwise *F*_ST_ values between the four sampled populations of *L. bonariensis* . (D) Violin plots of genome‐wide Tajima's *D*, calculated from 100 kb sliding windows throughout the *L. bonariensis* genome, with the dashed line representing a value of zero.

This differentiation of Chatham Island samples was maintained when PCA was repeated on a dataset created by intersecting the whole‐genome quality‐filtered SNPs and the SNPs identified by Harrop et al. (2020) using GBS (Figure 5), with PC1 explaining a similar percentage of variation at 12.7%. This intersected dataset contained 13,325 linkage‐pruned SNPs compared to the 6.69 million SNPs in the linkage‐pruned whole‐genome dataset.

**FIGURE 5:**
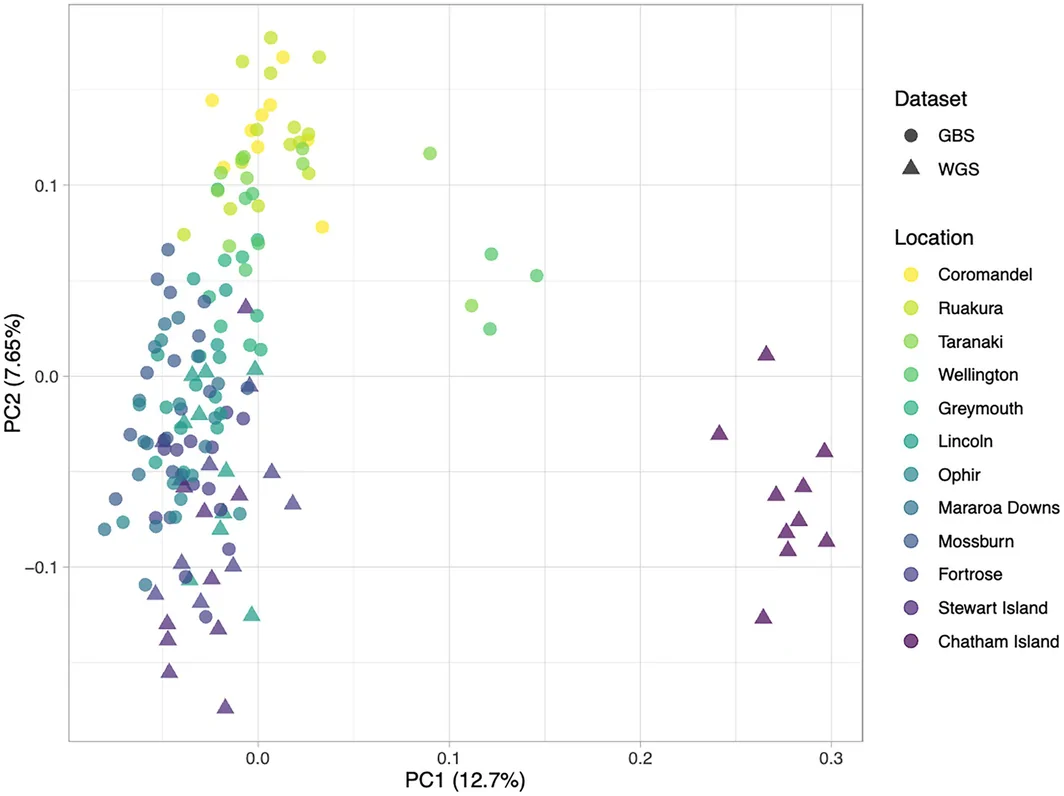
Principal Component analysis (PCA) of the merged dataset containing both whole‐genome sequenced (WGS) weevils and genotyping‐by‐sequencing (GBS) samples analysed previously by Harrop et al. (2020). This analysis was performed on 13,325 linkage‐pruned SNPs found in both the WGS and GBS datasets. Axes represent PC1 and PC2; colours represent collection location and shape represents the two datasets.

ADMIXTURE analysis was consistent with the genetic differentiation of Chatham Island samples identified by PCA (Figure 4B) at all values of *K* (i.e., the number of distinct genetic clusters inferred). At *K = 2*, which had the lowest cross‐validation (CV) error, the Chatham Island population forms a distinct cluster, clearly differentiated from the other three populations. Low proportions of the Chatham Island‐associated ancestry component were also inferred in some individuals from the other populations, consistent with some degree of shared genetic ancestry.

Cross‐validation error increased with each subsequent increase in *K* value (e.g., *K* = 3: 0.75047, *K* = 4: 0.86748). Values of *K* above two did not identify geographic differentiation at the population level, as was seen for Chatham Island at *K =* 2, but instead introduced additional population substructure. At *K = 3*, Lincoln and Stewart Island samples showed increased assignment to this third ancestry component (pink), whereas Fortrose remained predominantly associated with the prior component shared among the three non‐Chatham populations (yellow).

At *K = 4*, Chatham Island individuals were divided between two predominant ancestry components. This division corresponded with clustering along PC2 in the PCA when points are coloured by their predominant ADMIXTURE cluster (Figure S4). Low proportions of the two components predominant in the Chatham Island population were also inferred in some mainland and Stewart Island individuals, whereas the highest proportions of ancestry components in these non‐Chatham Island populations were not shared with Chatham Island individuals. Across *K* values from two to ten (Figure S5), neither mainland population nor Stewart Island showed geographic differentiation comparable to that observed for the Chatham Island population.

Pairwise *F*_ST_ test results (measuring genetic differentiation) of the *L. bonariensis* populations ranged between 0.017 and 0.104 (Figure 4C), with the highest values observed between the Chatham Island population and the other three populations (0.093–0.104), further supporting the Chatham Island's genetic differentiation. In contrast, pairwise *F*_ST_ values among mainland Fortrose, mainland Lincoln and Stewart Island (Figure 4C) were much lower (0.017–0.023), indicating greater genetic similarity. This pattern of greater differentiation of Chatham Island from other sampled populations may reflect differences in gene flow and/or demographic history between populations, including the magnitude of genetic bottleneck(s) associated with the introduction of *L. bonariensis* to New Zealand and/or its subsequent introduction to Chatham Island.

Estimates of Tajima's *D* values (Figure 4D) had a wide range encompassing both negative and positive values for all populations (−2.61 to 3.20). Mean Tajima's *D* values were consistently positive across all populations, with Chatham Island exhibiting the highest mean (0.76) compared to the other three populations (0.26 for Stewart Island, 0.40 for mainland Fortrose and 0.34 for mainland Lincoln). The positive distribution of Tajima's *D* values suggests a relative excess of intermediate‐frequency alleles, which was most pronounced in the Chatham Island population. This pattern may reflect differences in demographic history, including historical population bottleneck(s), but could also result from population structure or selection.

When plotted as a phylogenetic splits network, little genetic differentiation is seen among all sequenced individuals (Figure 6). Individuals sequenced from Stewart Island, Lincoln and Fortrose sit in mixed population clusters, and individuals sequenced from Chatham Island sit in a cluster of their own.

**FIGURE 6:**
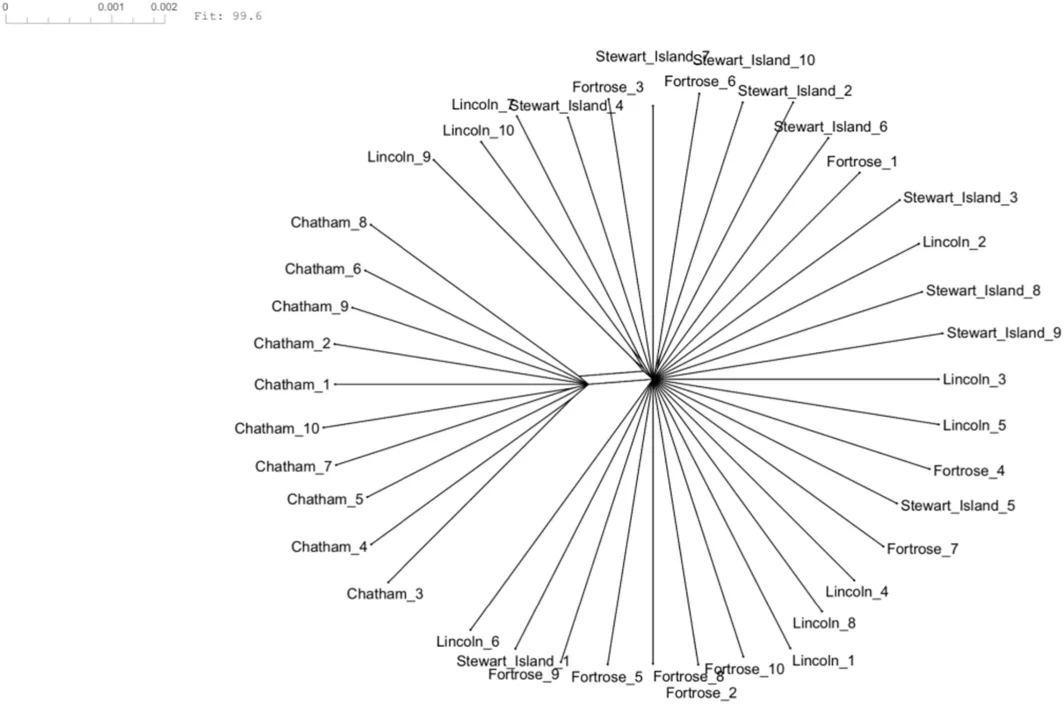
Split Network of *Listronotus bonariensis* collected on the Chatham and Stewart Islands, and Lincoln (Canterbury, New Zealand). The tree includes 40 *Listronotus bonariensis* genomes sequenced from three locations. The network was inferred with SplitsTree 6 using the Neighbour Net algorithms.

## 4 Discussion

### 4.1 Morphological Variation

The statistical analysis of the elytral lengths presented in the Results Section indicated that 16 measurements per sex per site reported small standard errors (0.02–0.03 mm). Thus, these error terms were minor relative to the ~0.3 mm between‐site differences, implying the sampling design is adequately powered for the effect claimed.

Although the *L. bonariensis* specimens collected from the Chatham Island and Stewart Island were smaller than those obtained from New Zealand's mainland productive pastures, the morphology of their reproductive structures showed no evidence of divergence. The observed body size variation was probably a plastic response to the nutritional environment. Arguably, since the island and mainland samples were not collected in the same year (Chatham Island 2014; Stewart Island and Lincoln 2016), inter‐annual variation in climate cannot be formally excluded as a contributor to the observed size differences. However, three lines of evidence indicate that this is unlikely to be the primary driver. First, the mainland marginal‐ecosystem specimens, collected across a range of earlier years, were as small as the island specimens, indicating that habitat quality rather than collection year aligns with body size. Second, the mean of Lincoln samples reported here is comparable to those measured at the same site in 1977–78 (Goldson 1979), indicating that elytral length at a given site is stable over decadal timescales. Finally, the Chatham Island climate is mild oceanic (Richards 1966) and therefore is less inter‐annually variable than in Canterbury. Work elsewhere has similarly shown that nutrient quality influences body size in other Curculionidae (e.g., Palmer 2002; Blake et al. 2011; Knapp and Uhnavá 2014). An implication of this finding, therefore, is that nearly all of the published *L. bonariensis* research has been conducted on the typically larger individuals sampled from highly productive pasture grasses, the morphology of which has been described by Goldson (1982) and Barker (1989b).

### 4.2 Genomic Variation

Genomic analyses in this contribution showed that the genomes of the Stewart Island‐collected specimens clustered with mainland samples irrespective of body sizes. Principal component and *F*_ST_ analyses indicate little differentiation between Stewart Island and the mainland populations, compared with the pronounced differentiation observed in the Chatham Island weevils. At higher values of *K*, Stewart Island individuals showed greater similarity in cluster proportions to Lincoln than Fortrose individuals; however, cluster assignment at these values of *K* cannot be interpreted as direct evidence of a specific demographic relationship. In general, these results indicate that there has been recent shared ancestry and/or gene flow between Stewart Island and mainland populations.

The genetics of the Chatham Island *L. bonariensis* population was found to be markedly different from the conspecific weevils collected elsewhere. This differentiation may reflect genetic drift associated with the establishment of the island population. In particular, a strong population bottleneck during or following colonisation, potentially involving a small number of founding individuals and thus a founder effect, could have resulted in the substantial divergence from the source population. Further, as discussed, the Chatham Island climate is classified as mild oceanic (Richards 1966), and is broadly similar to the southern part of New Zealand's North Island. Consequently, the pronounced genomic differentiation observed here does not necessarily require climatic adaptation as an explanation and could instead have arisen through demographic processes such as a severe establishment bottleneck and subsequent genetic drift. That the reproductive structures of the Chatham Island weevil population showed no morphological divergence from the other populations indicates that there was no incipient speciation. Such interpretation could be tested further by investigating possible genomic associations with climatic variables and/or looking for signatures of selective sweeps in the *L. bonariensis* genome.

### 4.3 Biogeographical Implications

Based on the findings of this study, it can be hypothesised that the source of the *L. bonariensis* population introduced to Chatham Island was mainland New Zealand, where it has only been established for < 125 years (Stewart et al. 2023). Although the observed differentiation of the Chatham Island population could be explained by an independent introduction from a different South American source than the mainland population (Williams et al. 1994), there is no record of any direct trade between South America and Chatham Island. However, given that the genetic diversity of *L. bonariensis* in South America is understudied, this cannot be completely ruled out. Conversely, and as alluded to above, the differentiation is probably the result of a strong founder effect based on a small subset of the mainland New Zealand *L. bonariensis* population being transported to the Chatham Islands in the early part of the 20th century. At this time, trade peaked between the two localities. Such a mode of establishment is supported by the identification of *L. bonariensis* in collections from the Chatham Islands as early as 1967 (MacFarlane 1979).

The potential for such unintentional human transportation of *L. bonariensis* to have occurred has been exemplified by McNeill et al. (2015), who found the far more recently introduced parasitoid *M. hyperodae* in the Chatham Island weevil population. The dispersive ability of the parasitoid adult wasp is minimal (e.g., McNeill et al. 2002; Cournoyer and Boivin 2005). Further, the adult weevil exhibits relatively short‐range and intermittent dispersal flight (Goldson 1981; Barker et al. 1989b; Goldson et al. 2023). Moreover, Goldson et al. (1999) found that *L. bonariensis* flight is greatly curtailed when parasitised by *M. hyperodae*. The implication here then is that the parasitoid was inadvertently introduced as a transported parasitised weevil from which, on arrival, a wasp larva pupated such that when it emerged it was in the vicinity of a population of the weevil.

Once on Chatham Island, the founding weevils probably rapidly invaded the islands' suitable habitat (mostly exotic Gramineae), aided by the species' high fecundity of up to 685 eggs per female (Kalvelage 1999) and intermittent flight (Goldson 1981; Barker et al. 1989b). Therefore, it can be conjectured that after weevil established in the available niches, further sporadic and inadvertent introductions of conspecifics to the Chatham Islands would probably not have been sufficient to displace the well‐established and widely distributed population. Based on recent sampling data, the *L. bonariensis* population has such high numbers on the Chatham Islands that it makes up 77% of all coleopteran species recovered from perennially grazed areas (McNeill et al. 2015).

This study provides insights into how island ecosystems may be modulated by anthropogenic influences. The marked genetic differentiation of the Chatham Island *L. bonariensis* population may reflect both the size of the founding population and the severity of the population bottleneck associated with its establishment. A severe initial bottleneck could have resulted in substantial genetic differentiation from mainland New Zealand through genetic drift, with subsequent geographic isolation and limited gene flow contributing to the maintenance of this differentiation. The Chatham Island weevil population therefore provides an opportunity to investigate how genetic variation established through a founding effect bottleneck may be maintained in an invasive population, and when and how speciation could occur.

## Author Contributions

**S. L. Goldson:** conceptualization (equal), data curation (supporting), formal analysis (equal), funding acquisition (lead), investigation (equal), methodology (equal), project administration (equal), resources (equal), validation (equal), visualization (equal), writing – original draft (lead), writing – review and editing (lead). **P. K. Dearden:** conceptualization (equal), data curation (equal), formal analysis (equal), funding acquisition (equal), investigation (equal), methodology (lead), project administration (equal), resources (equal), software (lead), supervision (equal), validation (equal), visualization (equal), writing – original draft (supporting), writing – review and editing (supporting). **K. R. Dainty:** conceptualization (equal), data curation (lead), formal analysis (lead), investigation (equal), methodology (equal), software (equal), visualization (lead), writing – original draft (supporting), writing – review and editing (equal). **S. N. Inwood:** conceptualization (equal), data curation (lead), formal analysis (lead), investigation (equal), software (equal), visualization (lead), writing – original draft (supporting), writing – review and editing (equal). **M. R. McNeill:** data curation (equal), investigation (equal), resources (lead). **J. W. M. Marris:** data curation (supporting), formal analysis (equal), methodology (supporting), resources (equal), writing – original draft (supporting), writing – review and editing (supporting). **F. Tomasetto:** data curation (equal), formal analysis (lead), investigation (equal), methodology (equal), visualization (equal). **B. I. P. Barratt:** conceptualization (equal), data curation (equal), resources (equal), validation (equal), writing – original draft (supporting), writing – review and editing (supporting).

## Funding

This work was supported by an AgResearch Science Prize awarded to S.L.G. and F.T. and the New Zealand MBIE Endeavour Grant 76508‐ENDSI‐UOO awarded to P.K.D.

## Conflicts of Interest

The authors declare no conflicts of interest.

## Supporting information


**Figure S1:** Dorsal and lateral aspects of the aedeagus of four males of *Listronotus bonariensis* from Blackmount mainland (designated NZ) and four from the Chatham Island (designated CI).
**Figure S2:** Ventral aspect of female tergite 8 of four females of *Listronotus bonariensis* from Blackmount mainland New Zealand (designated NZ) and four from the Chatham Islands (designated CI).
**Figure S3:** Principal Component analysis (PCA) of 40 sequenced *L. bonariensis* genomes. Principal components 1–20 are represented in individual dot plots. Colours represent collection location, based on 6,688,548 linkage‐pruned SNPs.
**Figure S4:** Principal Component analysis (PCA) of 40 sequenced *L. bonariensis* genomes, with samples coloured by the admixture cluster they have the highest proportion assigned to at each plotted value of *K*. Based on 6,688,548 linkage‐pruned SNPs, axes represent PC1 and PC2.
**Figure S5:** Extended population admixture analysis with values of *K* between two and ten. Each colour represents a distinct genetic cluster, and each vertical bar represents a different sample, with samples grouped based on their collection location.
**Table S1:** Sequencing statistics.

## Data Availability

The raw data, consisting of the morphological and genetic compositions of the populations studied, are publicly available and can be accessed at the following permanent repositories: morphological data are available in a Figshare repository: https://doi.org/10.6084/m9.figshare.28887539. Scripts used for the genomic analyses are available on Zenodo at https://doi.org/10.5281/zenodo.22089851.
