# Supplementary material for: Biogeography of Argentine Stem Weevil Invasion of Southern New Zealand and Two Offshore Islands
Source: Ecol Evol. 2026 Nov 3;16(11):e74483. doi: 10.1002/ece3.74483 (PMC13731724; doi:10.1002/ece3.74483)
Supplement: Supplementary file 1 [file ECE3-16-e74483-s001.docx]

**SUPPLEMENTARY MATERIAL**

**Supplementary Figure 1.** Dorsal and lateral aspects of the aedeagus of four males of *Listronotus bonariensis* from Blackmount mainland (designated NZ) and four from the Chatham Island (designated CI).

**Supplementary Figure 2.** Ventral aspect of female tergite 8 of four females of *Listronotus bonariensis* from Blackmount mainland New Zealand (designated NZ) and four from the Chatham Islands (designated CI).

**Supplementary Figure 3** Principal Component analysis (PCA) of 40 sequenced *L. bonariensis* genomes. Principal components 1-20 are represented in individual dot plots. Colours represent collection location, based on 6,688,548 linkage-pruned SNPs.

**Supplementary Figure 4** Principal Component analysis (PCA) of 40 sequenced *L. bonariensis* genomes, with samples coloured by the admixture cluster they have the highest proportion assigned to at each plotted value of K. Based on 6,688,548 linkage-pruned SNPs, axes represent PC1 and PC2.

**Supplementary Figure 5** Extended population admixture analysis with values of K between two and ten. Each colour represents a distinct genetic cluster, and each vertical bar represents a different sample, with samples grouped based on their collection location.

**Supplementary Table 1** Sequencing statistics

| **sample_name** | **Collection Location** | **% reads mapped** | **Mean map quality** | **Mean Coverage** | **Mean depth of coverage** | **Mean base quality** |
| --- | --- | --- | --- | --- | --- | --- |
| Chatham_10 | Chatham Island | 96.65 | 30.3377 | 80.2197 | 8.56489 | 35.5647 |
| Chatham_1 | Chatham Island | 93.81 | 30.2835 | 85.0308 | 17.6875 | 35.2025 |
| Chatham_2 | Chatham Island | 95.8 | 29.9479 | 84.1183 | 13.5353 | 35.0521 |
| Chatham_3 | Chatham Island | 95.74 | 29.598 | 80.5032 | 7.95077 | 34.9434 |
| Chatham_4 | Chatham Island | 96.25 | 30.1518 | 85.9175 | 23.2542 | 35.1978 |
| Chatham_5 | Chatham Island | 96.33 | 29.6334 | 83.3549 | 12.7198 | 35.2666 |
| Chatham_6 | Chatham Island | 96.37 | 29.5286 | 81.4781 | 9.35217 | 35.2159 |
| Chatham_7 | Chatham Island | 98.03 | 30.4074 | 81.6226 | 9.62727 | 35.2923 |
| Chatham_8 | Chatham Island | 97.95 | 30.163 | 79.5102 | 8.69516 | 35.6496 |
| Chatham_9 | Chatham Island | 96.86 | 30.7271 | 81.2437 | 10.4798 | 35.5807 |
| Fortrose_10 | Fortrose | 98.34 | 30.9235 | 81.1577 | 7.28535 | 35.2431 |
| Fortrose_1 | Fortrose | 98.39 | 31.4371 | 84.3013 | 12.2503 | 35.4393 |
| Fortrose_2 | Fortrose | 98.64 | 31.4148 | 82.7844 | 11.1307 | 35.4346 |
| Fortrose_3 | Fortrose | 98.8 | 31.8101 | 81.7571 | 9.19362 | 35.6246 |
| Fortrose_4 | Fortrose | 98.08 | 31.6674 | 82.5094 | 10.6966 | 35.5905 |
| Fortrose_5 | Fortrose | 98.4 | 31.3033 | 83.5837 | 11.3366 | 35.3409 |
| Fortrose_6 | Fortrose | 98.48 | 31.6641 | 82.7506 | 9.18546 | 35.2185 |
| Fortrose_7 | Fortrose | 98.45 | 31.2521 | 84.899 | 14.1865 | 35.3114 |
| Fortrose_8 | Fortrose | 98.73 | 31.6426 | 82.908 | 10.4728 | 35.511 |
| Fortrose_9 | Fortrose | 98.7 | 31.4575 | 80.9033 | 7.45269 | 35.4617 |
| Lincoln_10 | Lincoln | 97.93 | 31.0277 | 84.4024 | 14.438 | 35.5764 |
| Lincoln_10 | Lincoln | 97.9 | 31.3018 | 84.098 | 10.5552 | 35.2695 |
| Lincoln_2 | Lincoln | 87.54 | 28.2613 | 79.4384 | 7.87228 | 35.5428 |
| Lincoln_3 | Lincoln | 97.81 | 28.3855 | 78.0347 | 8.76419 | 35.5621 |
| Lincoln_4 | Lincoln | 98.42 | 31.8172 | 82.5736 | 8.87522 | 35.5386 |
| Lincoln_5 | Lincoln | 98.56 | 31.4963 | 80.9898 | 7.96897 | 35.353 |
| Lincoln_6 | Lincoln | 98.07 | 29.2667 | 82.8077 | 14.4698 | 35.2948 |
| Lincoln_7 | Lincoln | 98.71 | 31.5321 | 82.6551 | 10.9945 | 35.372 |
| Lincoln_8 | Lincoln | 97.95 | 30.6028 | 83.065 | 11.6684 | 35.6937 |
| Lincoln_9 | Lincoln | 98.97 | 31.3114 | 83.33 | 11.8557 | 35.383 |
| Stewart_Island_10 | Stewart Island | 94.1 | 29.6099 | 83.8529 | 10.8013 | 35.1219 |
| Stewart_Island_1 | Stewart Island | 96.58 | 31.139 | 81.6573 | 8.18224 | 35.3299 |
| Stewart_Island_2 | Stewart Island | 96.59 | 29.5153 | 80.9706 | 7.95472 | 35.4146 |
| Stewart_Island_3 | Stewart Island | 95.32 | 29.7813 | 81.0527 | 7.94865 | 34.8808 |
| Stewart_Island_4 | Stewart Island | 97.05 | 29.9965 | 84.4048 | 16.0891 | 35.5929 |
| Stewart_Island_5 | Stewart Island | 96.94 | 29.8058 | 83.8332 | 14.2796 | 35.7156 |
| Stewart_Island_6 | Stewart Island | 97.89 | 31.3985 | 84.6597 | 15.8366 | 35.582 |
| Stewart_Island_7 | Stewart Island | 97.49 | 31.5808 | 83.1972 | 11.7607 | 35.6846 |
| Stewart_Island_8 | Stewart Island | 96.59 | 29.3348 | 82.0997 | 9.5561 | 35.5183 |
| Stewart_Island_9 | Stewart Island | 93.17 | 28.5381 | 77.0628 | 4.92292 | 34.9611 |
